# Appendicitis in Children.—I

**Published:** 1911-04-01

**Authors:** O. L. Addison


### Hospital Clinics.

### APPENDICITIS IN CHILDREN.—I.

By O. L. ADDISON, F.R.C.S.

A Lecture at the Hospital for Sick Children, Great Ormond Street, W.C.

Appendicitis in children under twelve years of age, although less common than in adults, is nevertheless of frequent occurrence, and the number of cases admitted to this hospital is increasing year by year. Moreover, the gravity of the condition, and the rapidity with which the clinical picture may alter for the worse, makes a proper understanding of the various phases of this disease and of their appropriate treatment, of the very first importance.

Numerous cases have been recorded in children under the age of two years, and, indeed, several under the age of six months; but relatively few cases occur before the age of five years. Above this age the liability rapidly increases. In a small proportion of cases a direct history of injury may be obtained, in others a history of previous gastro-intestinal disturbance. More usually no cause is assigned. Fæcal concretions are found almost as frequently as in adults, but true foreign bodies, with the exception of thread-worms, are rare.

### "Bilious Attacks."

Sub-acute or chronic appendicitis is not uncommon. Here the child is generally the subject of chronic constipation, and has frequent colicky pains in the abdomen, often localised to the right side. This condition is not infrequently associated with so-called "bilious attacks," and may last for two or three years or even longer, but sooner or later it flares up into a typical attack of acute appendicitis.

In other cases, repeated "bilious attacks" are the only manifestations, the child being perfectly well in the intervals; in fact, it would hardly be going too far to say that appendicectomy would cure nearly all cases of "bilious attacks" in children.

Clinically, acute appendicitis may be divided into three groups. First, a mild form, in which the inflammation is either limited to the appendix itself, or at most causes the formation of a few adhesions. Secondly, appendicitis, with abscess formation. Thirdly, the acute fulminating attack, with profound toxæmia and general peritonitis.

### The Mild Type.

In the first type, the illness usually commences with a sudden attack of pain in the abdomen, referred at first to the umbilical region, later localised in the right iliac fossa. Vomiting generally begins shortly after the onset of the pain, and continues as a rule at intervals during the first twenty-four hours or even longer. Marked constipation is the rule, but sometimes there is diarrhœa. The temperature as a rule is raised two or three degrees, with a pulse-rate up to 120 or 130. Rigidity of muscles over the right iliac fossa, with tenderness, will be present. After the first twenty-four to forty-eight hours the pulse and temperature fall, the pain and tenderness diminish, and, if all goes well, by the third or fourth day nothing can be made out except perhaps a little tenderness in the right iliac fossa.

On the other hand, instead of improving, the patient's condition on the third day may be worse, the pulse-rate increasing, the temperature rising. The pain in the right iliac fossa increases, rigidity is more marked, and a mass may be felt, indicating that an abscess is in all probability present. In cases going on to abscess formation, the symptoms are often more severe from the start, and a rigor may be an early symptom.

### The Acute Type.

In the third type of case, the patient's condition from the first is grave; he assumes in a few hours an intensely toxic appearance, with sunken face of an earthy colour. The pulse is small, extremely rapid, and frequently out of all proportion to the temperature, which may be but slightly raised or even subnormal. So intense is the toxæmia in these cases that the child may be comatose or moribund within twenty-four hours. This type of case is fortunately rare, but it is most important to remember that this condition may develop with great rapidity from either of the two former types; in the first from sudden rupture of a distended appendix where sufficient protective adhesions have not formed, and in the second type either from the virulence of the organisms causing the abscess, or from sudden rupture of the abscess and diffusion of its contents in the peritoneal cavity.

### Difficulties of Diagnosis.

The diagnosis has to be made from a great variety of disorders and is often of the greatest difficulty:—

1. *In acute indigestion* a history or a recent excessive or indigestible meal is of value, as also is the presence of large particles of undigested food in the vomit. The pulse-rate and temperature may both be considerably raised, but there is no localised pain in the right iliac fossa, neither is there rigidity or tenderness in this region. Should the condition not have yielded to treatment within ten or twelve hours the case should be regarded with suspicion and treated as one of appendicitis.

2. *Pneumonia.* The onset may present almost insuperable diagnostic difficulties, for while there may be no definite physical signs in the lungs, there is often extreme pain referred to the abdomen, with hyperæsthesia and tenderness so great as to make palpation valueless. The character of the respiration with the disproportionate respiration pulse-rate will usually put one on one's guard, and in any case, after a delay of ten or twelve hours the signs in the lungs will have developed sufficiently to make the diagnosis clear.

3. *Obstruction.* The commencement of acute obstruction, although not unlike that of appendicitis, can usually be distinguished by the absence of local tenderness and the fact that the temperature and pulse-rate are not much affected at first. During the course of an attack of appendicitis signs of obstruction may develop either as a result of adhesions or from paralytic distension.

4. *Abdominal tuberculosis* may cause difficulty in several ways. Tuberculous glands (too small to be felt) in the ileo-cæcal angle not infrequently are the cause of attacks of colicky pains indistinguishable from those due to chronic appendicitis. In these cases, in which as a rule there is no local tenderness, the pain results from adhesions between the glands and the surrounding intestines. A local tuberculous infiltration and ulceration of the cæcum, a large mass of glands in the ileo-cæcal angle, or a tuberculous abscess in the iliac fossa, may all be found in conjunction with symptoms suggesting acute appendicitis, but as a rule the symptoms and local signs are less marked than would be the case were the mass due to an acute inflammation of the appendix. Acute infective peritonitis from perforation of a tuberculous ulcer in the intestine is not uncommon.

### General Peritonitis.

5. *Acute general peritonitis*, whether due to pneumococcal infection (a not uncommon cause in children) or to some other organism, naturally cannot be distinguished from that secondary to a perforated appendix. Pneumococcal peritonitis commonly begins with sudden pain about the umbilicus with constant vomiting and continuous diarrhœa, but the diagnosis of the cause is unimportant, treatment being the same whatever the organism may be.

6. *Enteric fever* may sometimes present itself with abdominal pain and tenderness localised in the right iliac fossa. The diagnosis may at first be impossible, but a history of malaise of some days' duration will probably be obtained, and an enlarged spleen or a positive Widal's reaction if present will settle the question.

7. *Intussusception.* Here the spasmodic nature of the attacks of pain, the abdomen being relaxed in the intervals, the presence of a mobile lump in the right iliac fossa, with passage of blood and mucus per rectum, make the diagnosis clear.

8. *Renal colic.* This is not common in children, and the pain and tenderness are most marked in the loin; the temperature is not as a rule raised more than one or two degrees, and blood quickly makes its appearance in the urine.

### A Common Condition.

9. *Spinal Caries and Hip Joint Disease.* Owing to the fact that flexion at the hip joint is common in all three conditions, mention is made in text-books of the difficulty in diagnosis between these two diseases and appendicitis. Hip joint disease should cause no difficulty, because here the flexion is due either to a general spasm of the muscles about the hip joint, or to ankylosis, in either case causing immobility of the joint. In spinal caries, as in appendicitis, the flexion of the hip is due to spasm of the ilio-psoas, movements of the hip not involving stretching of that muscle (*e.g.* flexion and external rotation) being free. Ilio-psoas spasm may be produced by inflammation spreading from any of the neighbouring structures, whether vertebræ, pelvic bones, kidney, iliac glands or appendix, and it is always necessary to bear this fact in mind in a doubtful case in which ilio-psoas spasm is a prominent feature.

The foregoing list of conditions in which confusion may arise is a formidable one, but in the majority of cases the difficulty will disappear if the patient be carefully watched for from ten to twelve hours. If his condition be too grave to allow of this delay, operation would be the correct treatment.

### Temperature in Childhood.

I have avoided laying stress on the temperature as a diagnostic feature in these cases, as in children the temperature rises so readily with the slightest cause that it is a most unreliable guide to the gravity of the condition. The state and rate of the pulse are by far the best indications of the progress of the case. However, a temperature steadily rising after the third or fourth day, a degree or more each day, is a fairly certain sign that an abscess is forming. A blood count should always be made where possible, a leucocytosis of over 12,000, or, better still, an increasing leucocytosis in successive counts, pointing strongly to the presence of pus.

### An Occasional Condition.

The pain varies very greatly, both in severity and locality, and although mostly sudden in onset, is by no means always so, often increasing gradually in severity. The pain is at first usually at the umbilicus, later in the right iliac fossa, but may be general over the lower half of the abdomen, or even in the left iliac fossa. Hyperæsthesia of the skin over the appendix region is occasionally present, but is not, as it has been often said to be, consequent upon any particular pathological condition of the appendix.

Urinary symptoms are frequent, and an acute attack of pain exactly simulating renal colic, and followed by hæmaturia, is sometimes met. Retention, or more frequently difficulty in micturition, occurs, with severe pain during the act referred to the end of the penis. These symptoms invariably point either to adhesions at the back of the bladder or to a collection of pus in Douglas' pouch.

### Constipation and Distention.

Obstinate constipation is the rule, but profuse diarrhœa is not infrequent in the early stages, or as the result of an aperient, and in the more chronic forms of the disease a definite mucous colitis from irritation of the large bowel is not uncommon. Paralytic distension or obstruction from adhesions also occurs. A sudden free discharge of mucus in the motions often immediately precedes the rupture of an abscess into the rectum.

(*To be continued.*)

